# ST-GCP: a graph convolutional network model with contrastive consistency and permutation for spatial transcriptomics

**DOI:** 10.1093/bib/bbaf643

**Published:** 2025-12-05

**Authors:** Yajie Meng, Yongkang Wang, Cheng Guo, Xianfang Tang, Zilong Zhang, Feifei Cui, Xiangzheng Fu, Quan Zou, Xu Lu, Junlin Xu

**Affiliations:** School of Computer Science and Artificial Intelligence, Wuhan Textile University, No. 1 Sunshine Avenue, Jiangxia District, Wuhan, Hubei 430200, China; School of Computer Science and Artificial Intelligence, Wuhan Textile University, No. 1 Sunshine Avenue, Jiangxia District, Wuhan, Hubei 430200, China; School of Computer Science and Technology, Wuhan University of Science and Technology, 2 Huangjiahu West Road, Hongshan District, Wuhan, Hubei 430065, China; School of Computer Science and Artificial Intelligence, Wuhan Textile University, No. 1 Sunshine Avenue, Jiangxia District, Wuhan, Hubei 430200, China; School of Computer Science and Technology, Hainan University, No. 58 Renmin Avenue, Meilan District, Haikou, Hainan 570228, China; School of Computer Science and Technology, Hainan University, No. 58 Renmin Avenue, Meilan District, Haikou, Hainan 570228, China; School of Chinese Medicine, Hong Kong Baptist University, 7 Baptist University Road, Kowloon Tong, Hong Kong, SAR 999077, China; Institute of Fundamental and Frontier Sciences, University of Electronic Science and Technology of China, No. 2006 Xiyuan Avenue, High-Tech Zone, Chengdu, Sichuan 610054, China; School of Computer Science, Guangdong Polytechnic Normal University; Guangdong Provincial Key Laboratory of Intellectual Property & Big Data, No. 293 Zhongshan Avenue West, Tianhe District, Guangzhou, Guangdong 510665, China; School of Computer Science and Technology, Wuhan University of Science and Technology, 2 Huangjiahu West Road, Hongshan District, Wuhan, Hubei 430065, China

**Keywords:** spatial transcriptomics, permutation, clustering, spatial domain identification

## Abstract

Spatial transcriptomics (STs) technology is a powerful technique that simultaneously preserves gene expression profiles and spatial information, enabling deeper exploration of tissue organization and function. However, many existing computational approaches often rely on labeled ST data and overlook the rich spatial information, resulting in limited representations and suboptimal clustering. In this paper, we propose ST-GCP, a self-supervised graph representation learning framework for ST data, which incorporates a structure-feature perturbation mechanism. First, ST-GCP applies feature-level random permutation of the gene expression matrix and random edge dropout in the spatial neighbor network, creating two complementary augmented graph views of ST data. ST-GCP then employs a two-layer graph convolutional network (GCN) encoder-decoder to extract spatial representations and reconstruct gene expression. Finally, a cosine-similarity-based contrastive objective aligns the view-specific representations, and the overall loss jointly optimizes reconstruction fidelity and contrastive consistency, thereby coupling graph topology with transcriptomic profiles in a shared low-dimensional space. Experimental results on multiple ST datasets demonstrate that ST-GCP can uncover biologically meaningful patterns, such as tumor heterogeneity, brain developmental architecture, and cellular developmental trajectories.

## Introduction

Single-cell RNA sequencing (scRNA-seq) has enabled high-precision dissection of intercellular transcriptional heterogeneity at single-cell resolution [1, 2]. However, the dissociation of tissues during scRNA-seq leads to a loss of spatial context, thereby limiting the ability to elucidate spatially regulated biological mechanisms. Spatial transcriptomics (STs) bridges this gap by capturing the spatial topological relationships of tissue microenvironments, offering a novel perspective for revealing the spatial regulatory mechanisms of gene expression [3, 4]. Current mainstream STs technologies, including 10x Visium [5], Slide-seqV2 [6], Stereo-seq [7], STARmap [8], osmFISH [9], and seqFISH+ [10], not only support the construction of spatial transcriptome atlases at single-cell resolution but also facilitate tasks such as deciphering spatial neighborhoods and dynamically tracking developmental trajectories [11, 12].

Identifying spatial domains with similar expression patterns remains one of the key challenges in STs data analysis. Taking the labeled brain tissue of adult mouse as an example, the laminar structure of specific cerebral cortices is closely associated with their biological functions. Cells within different cortical layers often exhibit differences in morphological characteristics, gene expression patterns, and biological functions [13, 14]. Consequently, various clustering methods that leverage spatial information to identify spatial domains have been developed. Based on annotated datasets, such as the human dorsolateral prefrontal cortex (DLPFC) profiled by the 10x Visium platform and the mouse visual cortex captured by the STARmap platform, traditional clustering methods like K-means [15, 16], Louvain [17, 18], and Leiden [19] are computationally efficient. However, they often produce fragmented spatial domains when applied to data with large inter-spot distances or low spatial resolution, making it difficult to accurately identify continuous spatial structures. Moreover, they struggle to capture the complex spatial dependencies inherent in the high-dimensional and noise-prone ST data, thereby limiting their ability to accurately delineate functionally heterogeneous regions within the tissue microenvironment.

In recent years, clustering algorithms that model spatial dependencies have markedly improved the accuracy of spatial domain identification by leveraging the functional similarity among spatially adjacent spots. Current research methods mainly evolve along three technical paths. The first involves statistical inference methods based on probabilistic graphical models, such as Giotto [20], which constructs a hidden Markov random field (HMRF) to capture the potential associations between adjacent spots, and BayesSpace [21], which introduces spatial prior constraints through a Bayesian framework to improve clustering consistency. The second path involves representation learning methods based on deep graph neural networks. Representative methods include SEDR [22], which uses a dual auto-encoder architecture to process gene expression and spatial topology separately, STAGATE [23] introduces a cell type-aware network and graph attention mechanism to evaluate neighborhood similarity and identify spatial domains, then SpaceFlow [24] enhances the discriminability of latent representations through deep graph neural networks with contrastive learning strategies. stAA [25] incorporates global graph information into cell embeddings through an adversarial graph autoencoder to enhance model performance. STMSGAL [26] utilizes a graph attention autoencoder with multiscale deep subspace clustering to generate latent spot representations for spatial domain identification. The third path includes multimodal information fusion methods. For example, stLearn [27] combines histological image features with spatial adjacency relationships, and SpaGCN [28] constructs weighted graphs based on Graph Convolutional Network (GCN) [29] models to integrate data such as gene expression, spatial coordinates, and pathological images. Although these existing methods have made progress in feature representation, they mainly rely on large labeled ST data and show limited sensitivity to gradient changes in boundary-region spots, resulting in nonsmooth domain transitions. Additionally, the absence of random mechanisms for modeling complex spatial relationships significantly hinders their ability to resolve fine-grained tissue heterogeneity.

In this paper, we propose a GCN framework, ST-GCP, which utilizes random permutation, random edge dropout, and contrastive loss to perform spatial clustering while reducing reliance on labeled ST data. Firstly, ST-GCP constructs contrastive views by applying random permutation to the gene expression matrix and performing random edge dropout on the spatial neighbor graph. ST-GCP then leverages hierarchical graph convolutional modules to extract spatial features from permuted graphs, followed by latent space mapping through a graph autoencoder. To jointly optimize structural and representational consistency, we design a multi-objective loss function that combines reconstruction loss with contrastive loss. This formulation ensures topological alignment between the input and reconstructed graphs, as well as feature consistency between the two reconstructed matrices. Benefiting from this design, ST-GCP demonstrates enhanced clustering robustness and strong generalizability across various STs platforms and resolutions. We evaluate ST-GCP on complex tissue architectures such as the human cerebral cortex and mouse visual cortex, profiled using platforms with different spatial resolutions including 10x Visium, Slide-seqV2, and STARmap. Furthermore, we benchmark ST-GCP against seven representative methods. Experimental results show that ST-GCP outperforms existing methods in spatial domain identification and provides superior biological interpretability in characterizing tissue heterogeneity.

## Materials and Methods

### Data preprocessing

The ST-GCP framework takes gene expression profiles and spatial coordinate data as inputs. Firstly, the top 3000 highly variable genes (HVGs) are selected using SCANPY [19]. Subsequently, the data are normalized and log-transformed. Finally, a scaling step is applied to constrain the range of expression values. Additionally, an undirected adjacency graph $G=\left(V,E\right)$ is constructed, where the vertex set $V$ represents spatial spots and the edge set $E$ denotes spatial adjacency relationships. The adjacency matrix, denoted as $A\in{R}^{N_{spot}\times{N}_{spot}}$, is defined as:


(1) \begin{equation*} {A}_{ij}=\left\{\begin{array}{l}1,\kern0.75em if\ {\left\Vert{x}_i-{x}_j\right\Vert}_2\le r\\{}0,\kern0.75em otherwise,\end{array}\right. \end{equation*}

Where ${x}_i$ and ${x}_j$ represent the the two-dimensional spatial coordinates of spot $i$ and $j$, respectively, and the threshold $\mathrm{r}$ is set to 150.

### Structure-feature perturbation mechanism

To support the joint optimization framework of feature reconstruction and contrastive consistency, we adopt two data augmentation strategies: random permutation of the gene expression matrix and random edge dropout on the spatial neighbor network. Specifically, for a given spatial neighborhood graph $G=\left(V,E\right)$ and its normalized gene expression matrix $X\in{R}^{n\times d}$, we generate a perturbed feature matrix by applying permutation that shuffles the rows of $X$ while preserving the original graph topology. A random permutation operator $\varepsilon$ is defined over the node indices. The permuted matrix ${X}^{\prime}\in{R}^{n\times d}$ is constructed as:


(2) \begin{equation*} {X}^{\prime }=X\left[\varepsilon, :\right]=\left[\begin{array}{c}{x}_{\varepsilon (1)}\\{}{x}_{\varepsilon (2)}\\{}\vdots \\{}{x}_{\varepsilon (n)}\end{array}\right]\in{R}^{n\times d} \end{equation*}

where ${x}_{\varepsilon \left(\mathrm{i}\right)}$ denotes the feature vector corresponding to the $i- th$ row in the original matrix.

Random edge dropout is a strategy for probabilistically removing edges from the original graph structure before training, which randomly sparsifying the original graph with a fixed drop rate. Specifically, a subset of edges is randomly dropped from the original edge set $E$, resulting in a sparsified edge set ${E}^{\prime}\subseteq E$ that defines the structure of the augmented graph. We apply random edge dropout to construct a sparsified adjacency structure ${A}^{\prime }$, which is shared across both contrastive views and is defined as:


(3) \begin{equation*} {A}^{\prime }= DropEdge\left(A,p\right) \end{equation*}

Where $p$ denotes the edge dropout rate, with the default value set to 0.1, and it is kept fixed during experiments without being adjusted according to training epochs or dataset scale.

Meanwhile, the node feature matrix ${X}^{\prime}\in{R}^{n\times d}$ is perturbed via row-wise random permutation, resulting in an augmented feature matrix ${X}^{\prime }$. The two contrastive graph views are then defined as:


(4) \begin{equation*} {G}_1^{\prime }=\left(V,{A}^{\prime },X\right) \end{equation*}

(5) \begin{equation*} {G}_2^{\prime }=\left(V,{A}^{\prime },{X}^{\prime}\right) \end{equation*}

Where ${A}^{\prime }$ is the edge-dropped adjacency structure (represented as an edge index set). Sharing the same adjacency structure ${A}^{\prime }$ between the two views preserves the intrinsic spatial neighborhood information, ensuring that the spatial relationships between neighboring cells are maintained.

### Graph convolutional network

After data augmentation, the third core component of ST-GCP is a graph convolutional neural network. The goal of the graph encoding stage is to jointly model the augmented gene expression matrix and spatial neighbor network through a graph neural network, enabling the learning of biologically meaningful low-dimensional representations. Here, we employ a two-layer GCN [29] to process the augmented graph ${G}^{\prime }=\left(V,{A}^{\prime },{X}^{\prime}\right)$, which effectively preserves both local topological structure and global expression patterns in STs data. The design fully leverages the advantages of GCN in preserving local topological structures and global feature distributions, which are particularly critical for deciphering spatial distribution patterns in ST data.

Specifically, for the augmented graph ${G}_1^{\prime }$, the hidden feature vector ${h}_i^{(L)}$ of node $i$ at layer $L$ is calculated as follows:


(6) \begin{equation*} {H}_1^{(1)}=\sigma \left({\hat{A}}^{\prime }X{W}_1\right) \end{equation*}

(7) \begin{equation*} {H}_1^{(2)}=\sigma \left({\hat{A}}^{\prime }{H}^{(1)}{W}_2\right) \end{equation*}

Meanwhile, for the augmented graph ${G}_2^{\prime }$, the hidden feature vector ${h}_i^{(L)}$ of node $i$ at layer $L$ is calculated as follows:


(8) \begin{equation*} {H}_2^{(1)}=\sigma \left({\hat{A}}^{\prime }{X}^{\prime }{W}_1\right) \end{equation*}

(9) \begin{equation*} {H}_2^{(2)}=\sigma \left({\hat{A}}^{\prime }{H}^{(1)}{W}_2\right) \end{equation*}

where ${\hat{A}}^{\prime }={\hat{D}}^{-1/2}{\hat{A}}^{\prime }{\hat{D}}^{-1/2}$ is the symmetrically normalized adjacency matrix with self-loops $\left({\hat{A}}^{\prime }={A}^{\prime }+I\right)$, ${W}_1$ and ${W}_2$ are trainable weight matrices, $\sigma \left(\cdotp \right)$ is a nonlinear activation function (e.g. ReLU), ${H}^{(2)}$ is the learned low-dimensional embedding. Additionally, the two augmented graphs share the same weight parameters ${W}_1$ and ${W}_2$.

For the two augmented graphs ${G}_1^{\prime }$ and ${G}_2^{\prime }$, the low-dimensional embeddings obtained through two-layer information GCN encoding are:


(10) \begin{equation*} {H}_1={GCN}_{enc}\left({A}^{\prime },X\right) \end{equation*}

(11) \begin{equation*} {H}_2={GCN}_{enc}\left({A}^{\prime },{X}^{\prime}\right) \end{equation*}

In the graph decoding stage, ${H}_1$ and ${H}_2$ are reconstructed through a two-layer GCN to obtain reconstructed feature matrices. Based on the implemented operations, the computation can be summarized by the following formula:


(12) \begin{equation*} {\hat{X}}_1={GCN}_{dec}\left({A}^{\prime },{H}_1\right)= dec\_ conv2\left( dec\_ conv1\left({H}_1,{A}^{\prime}\right),{A}^{\prime}\right) \end{equation*}

(13) \begin{equation*} {\hat{X}}_2={GCN}_{dec}\left({A}^{\prime },{H}_2\right)= dec\_ conv2\left( dec\_ conv1\left({H}_2,{A}^{\prime}\right),{A}^{\prime}\right) \end{equation*}

These reconstructed features ${\hat{X}}_1$ and ${\hat{X}}_2$ are used to compute reconstruction loss and contrastive consistency loss in the final objective.

### Loss function

The ST-GCP adopts a joint optimization strategy that simultaneously optimizes feature reconstruction and contrastive representation learning to enhance model performance. Specifically, the total loss consists of two components: reconstruction loss and contrastive loss, which are jointly minimized to guide both accurate feature reconstruction and robust representation learning.

The reconstruction loss quantifies the discrepancy between the original feature matrix $X$ and the reconstructed feature matrices ${\hat{X}}_1$ and ${\hat{X}}_2$, generated from the two augmented graph views. Specifically, this loss is evaluated by computing the mean squared error (MSE) [30] between the original feature matrix and the reconstructed feature matrix:


(14) \begin{equation*} {L}_{rec}=\frac{1}{n}\sum_{i=1}^n\left({\left|X-{\hat{X}}_1\right|}_2^2+{\left|{X}^{\prime }-{\hat{X}}_2\right|}_2^2\right) \end{equation*}

where $n$ denotes the number of nodes in the graph. Minimizing this loss ensures that the reconstructed features retain fidelity to the original gene expression profiles.

On the other hand, the contrastive loss facilitates the model to learn consistent feature representations between the two reconstructed graphs. To compute this loss, the reconstructed feature matrices ${\hat{X}}_1$ and ${\hat{X}}_2$ are passed through global average pooling to obtain embeddings:


(15) \begin{equation*} re{c}_1= global\_ mean\_ pool\left({\hat{X}}_1\right) \end{equation*}

(16) \begin{equation*} re{c}_2= global\_ mean\_ pool\left({\hat{X}}_2\right) \end{equation*}

Where $re{c}_1$ and $re{c}_2$ denote the global average pooled embeddings derived from the reconstructed feature matrices.

The contrastive loss is designed to minimize the differences between the two reconstructed graphs, which is achieved by minimizing the cosine similarity between the reconstructed features $re{c}_1$ and $re{c}_2$:


(17) \begin{equation*} {L}_{cl}=\frac{re{c}_1\bullet re{c}_2}{\left\Vert re{c}_1\right\Vert \left\Vert re{c}_2\right\Vert } \end{equation*}

Where ${L}_{cl}$ denotes the contrastive loss. This alignment objective quantifies the similarity between the two views, contributing to enhanced robustness against perturbations and helping to prevent representation collapse.

The overall loss function integrates ${L}_{rec}$ and ${L}_{cl}$ as: 


(18) \begin{equation*} L={L}_{rec}-{\lambda L}_{cl} \end{equation*}

**Figure 1:**
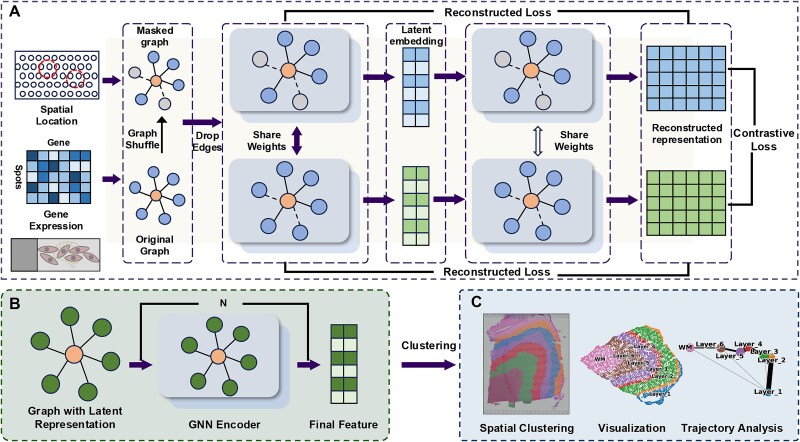
Architecture of ST-GCP. (A) ST-GCP first constructs a spatial neighbor network through a preset radius, introduces structure-feature perturbation strategy to random permutation the gene expression matrix, and combines random edge dropout to generate two contrastive views to enhance the model’s robustness and generalization. The model then employs a graph convolutional neural network to extract low-dimensional latent representations of nodes and optimizes the feature space through reconstructed loss and contrastive loss. (B) Nodes are processed through the GNN encoding layer to obtain the final features. (C) the final low-dimensional features obtained by ST-GCP are used for spatial clustering analysis, including spatial domain identification and trajectory inference analysis.

The integration of ${L}_{rec}$ and ${L}_{cl}$, $\lambda$ is a weighting parameter used to control the relative importance of the two losses. By default, both are assigned equal weights of 1. The final cluster assignment is obtained via the mclust algorithm. The full workflow is illustrated in Fig. 1.

### Evaluation metrics

For ST data with known ground-truth labels, the study employs the ARI [31] and NMI [32] as a dual-index validation system. ARI evaluates clustering performance by calculating the consistency between sample ground-truth labels and predicted clustering results, while accounting for the impact of random consistency. This index ranges from −1 to 1, with values closer to 1 indicating higher consistency between clustering results and true biological structure. The ARI calculation is expressed as follows:


(19) \begin{equation*} ARI=\frac{RI-E\left[{RI}_{random}\right]}{\max (RI)-E\left[{RI}_{random}\right]} \end{equation*}

where the unadjusted Rand index is $RI=\frac{a+b}{C_n^2}$, where ${C}_n^2$ indicates the total number of possible pairs. Here, $a$ denotes the number of sample pairs that are correctly assigned to the same cluster, and $b$ denotes the number of sample pairs that are correctly assigned to different clusters. $E\left[{RI}_{random}\right]$ indicates the expected $RI$ based on random labeling.

NMI quantifies the statistical dependence between clustering labels and true categories by calculating the information overlap degree, with its normalized output ranging from 0 to 1. When the NMI approaches 1, it indicates that the clustering division achieves maximum information alignment with the true distribution. Then, when it approaches 0, it suggests that the clustering and true labels are nearly independent. Assuming $C$ represents the clustering results and $Y$ represents the ground-truth labels, the NMI calculation is expressed as follows:


(20) \begin{equation*} NMI=\frac{I\left(Y;C\right)}{\sqrt{H(Y)\bullet H(C)}} \end{equation*}

where $H(Y)$ and $H(C)$ represent the entropies of the predicted partition and the true partition, respectively. $I\left(Y;C\right)$ represents mutual information, which can be regarded as the amount of information about one random variable contained in another random variable. A higher NMI score denotes better performance.

### Clustering

Spatial domain identification is performed based on the low-dimensional embeddings learned by ST-GCP. When the number of clusters is predefined, the mclust algorithm is used to cluster the embedding features. We predefined different numbers of clusters for the real datasets generated from various platforms. Specifically, in the DLPFC dataset, based on manual annotation labels, the number of clusters was set to seven for eight slices and five for the remaining four slices. For the other datasets, the numbers of clusters were set as follows: 20 for the human breast cancer dataset, 11 for the mouse olfactory bulb dataset from the Slide-seqV2 platform, seven for the Stereo-seq dataset, 15 for the mouse brain dataset from the 10x Visium platform, and seven for the mouse visual cortex dataset from the STARmap platform. Each cluster consists of spots with similar gene expression patterns and adjacent spatial locations, corresponding to a spatial domain. Meanwhile, UMAP is used to visualize the clustering results to intuitively present the distribution characteristics of spatial domains [33].

## Results

### Overview of the ST-GCP framework

The ST-GCP framework takes spatial coordinates and gene expression matrices from ST data as input. It first constructs a spatial neighbor graph using a predefined radius to capture the topological relationships between spots. Next, ST-GCP generates augmented graphs for contrastive consistency by applying random permutation to the gene expression matrix and performing random edge dropout based on a probabilistic strategy. Subsequently, a two-layer GCN [29] is employed to jointly model the enhanced graph structure and gene expression features. Through hierarchical information propagation, the GCN captures multiscale spatial patterns and generates low-dimensional latent representations that integrate both local neighborhood information and global tissue architecture. ST-GCP is optimized using a multi-objective function fusing reconstruction loss and contrastive loss. The reconstruction loss enforces consistency between original and reconstructed features via MSE, and the contrastive loss enhances the alignment between the two reconstructed feature representations generated by the graph decoder through cosine similarity (Fig. 1A). For spatial domain identification, ST-GCP applies the learned latent embeddings to the mclust [16] clustering algorithm. The resulting clusters can be further analyzed through downstream tasks, such as trajectory inference using PAGA (Fig. 1B and 1C). Additionally, ST-GCP constructs adjacency graphs based on Euclidean distances [23, 34] to maintain spatial continuity and leverages random permutation to increase sensitivity to local structure. The strategies reduce dependence on labeled data and provide consistent performance across different ST platforms and resolutions. The framework supports the analysis of tissue heterogeneity at varying spatial scales.

### Datasets

To comprehensively evaluate the performance of ST-GCP, we utilized datasets from four different platforms (Table 1). Firstly, the human DLPFC dataset, generated using the 10x Visium platform, consists of 12 consecutive spatial tissue slices, each containing between 3460 and 4789 spatial spots. All slices are manually annotated and have been widely adopted as benchmark data in the field of STs [35]. Additionally, we included a human breast cancer dataset (Block A, Section 1), also generated with the 10x Visium platform, consisting of 3798 spots with annotations distinguishing cancerous and noncancerous regions [22]. To further validate the generalizability of ST-GCP across diverse biological contexts, we incorporated two datasets of adult mouse brain dataset (mouse brain posterior and mouse brain coronal sections) [36], enabling evaluation in both tumor microenvironments and structurally complex brain regions. To evaluate the model performance under high-resolution spatial settings, we utilized a Slide-seqV2-based mouse olfactory bulb dataset (10 μm resolution, 21 724 spots) [37] and a Stereo-seq-based stereo-sequential mouse olfactory bulb dataset (14 μm resolution, 19 109 spots), both of which offer near single-cell or subcellular resolution and are ideal for fine-scale spatial structure delineation [38]. Finally, to further evaluate cross-platform robustness and biological interpretability, we utilized a STARmap-based mouse visual cortex dataset containing gene expression profiles of 1020 genes across 1207 cells, accompanied by cell type annotations [8]. All datasets include standardized gene expression matrices along with corresponding spatial coordinates. Additional details on the dataset configuration are provided in the Supplementary Material.

**Table 1:** Summary of all datasets in this study.

**Platform**	**Tissue**	**Section**	**Spots**	**Genes**
10x Visium	DLPFC	151 507	4226	20 494
		151 508	4384	20 083
		151 509	4789	20 732
		151 510	4634	20 475
		151 669	3661	20 583
		151 670	3498	20 338
		151 671	4110	21 037
		151 672	4015	10 725
		151 673	3639	21 267
		151 674	3673	21 897
		151 675	3592	20 783
		151 676	3460	20 806
	Human breast cancer	Block A Section 1	3798	36 601
	Adult mouse brain	Mouse brain posterior	3355	32 285
		Mouse brain coronal	2903	32 285
Slide-seqV2	Mouse olfactory bulb	Puck_200127_15	21 724	11 750
Stereo-seq	Mouse olfactory bulb		19 109	27 106
STARmap	Mouse visual cortex	X	1207	1020

### ST-GCP improves the identification of spatial domains in spatial clustering on human DLPFC data

**Figure 2:**
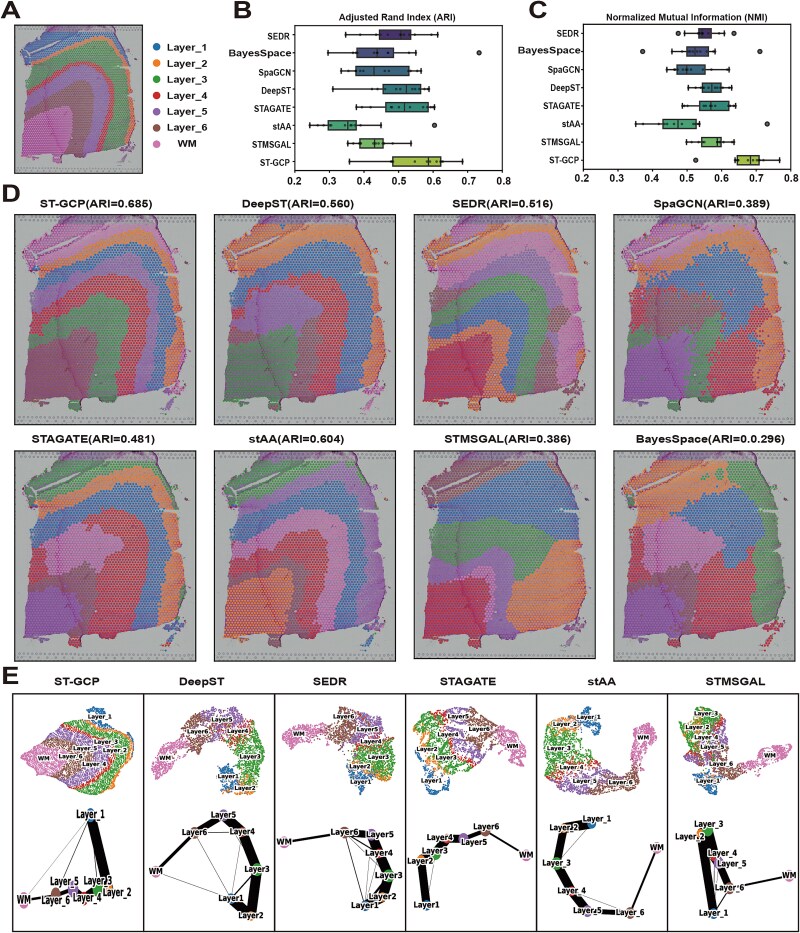
Results of ST-GCP and other benchmark methods tested on DLPFC dataset. (A) Ground-truth partition annotations of each layer of the cerebral cortex and WM in DLPFC section 151 674. (B) Boxplots of average ARI values across 12 samples for eight methods (SEDR, BayesSpace, SpaGCN, DeepST, STAGATE, stAA, STMSGAL, and ST-GCP). Each method is run 10 times to eliminate the influence of randomness. (C) Boxplots of average NMI values across 12 samples for eight methods. (D) Clustering results of SEDR, BayesSpace, SpaGCN, DeepST, STAGATE, stAA, STMSGAL, and ST-GCP on section 151 674. (E) UMAP visualization and PAGA graphs generated from the latent embedding results of eight methods on section 151 674.

We assessed the spatial clustering performance of ST-GCP using the human DLPFC dataset. This widely used benchmark dataset comprises 12 STs samples, sourced from the LIBD Brain Bank project [39] and accompanied by manually curated ground truth annotations. Specifically, four samples (151 669, 151 670, 151 671, and 151 672) are annotated with five spatial domains, while the remaining eight tissue slices are labeled with seven spatial regions—corresponding to six cortical layers and the white matter (WM) region. To systematically evaluate model performance, we selected seven current mainstream spatial clustering algorithms as comparison benchmarks, including SEDR [22], BayesSpace [21], SpaGCN [28], DeepST [40], STAGATE [23], stAA [25] and STMSGAL [26]. As part of the experimental design, sample 151 674 (with its ground truth spatial distribution shown in Fig. 2A) is used as a representative case to compare the seven methods and demonstrate the clustering advantage of ST-GCP.

The experimental results show that ST-GCP exhibits performance advantages in both spatial domain parsing accuracy and robustness. As shown in Fig. 2B and 2C, Adjusted Rand Index (ARI) [31] and Normalized Mutual Information (NMI) [32] are used to evaluate the clustering results of 12 samples, and these two indicators intuitively reflect the similarity between the predicted labels and the manually annotated ground truth [41]. In the test on section 151 674, ST-GCP achieved the highest ARI and NMI scores, and its spatial domain division was highly consistent with the ground truth annotations (Fig. 2D), highlighting its robustness in the task of parsing spatial domains. Among the comparative methods, the clustering consistency indicators of BayesSpace, stAA, and STMSGAL were generally low, while the average performance of the four methods SEDR, SpaGCN, DeepST, and STAGATE was relatively close and moderate. The manually annotated spatial domain map of sample 151 674 is shown in Fig. 2A, and the spatial domain identification results of the seven methods on this section are summarized in Fig. 2D. Each method used seven preset cluster numbers in this sample to ensure the fairness of the evaluation. Specifically, ST-GCP achieved parsing results of ARI = 0.685 and NMI = 0.767 on this sample, significantly better than the other methods, and the shape of the spatial domain division was more consistent with the true tissue structure. In comparison, stAA (ARI = 0.604, NMI = 0.732), DeepST (ARI = 0.560, NMI = 0.596), and SEDR (ARI = 0.516, NMI = 0.536) still had obvious regional boundary fuzziness in their spatial parsing results. The ARI of BayesSpace, SpaGCN, STAGATE, and STMSGAL on this sample was lower than 0.5, and the NMI value was below 0.6.

 The detailed ARI values of the eight methods across 12 spatial slices are presented in Supplementary Fig. S1. By comparing these ARI values, ST-GCP achieved the highest scores in seven spatial slices. Among the eight STs clustering methods evaluated in this study, all except BayesSpace are based on graph autoencoders. Overall, ST-GCP achieved the best clustering performance, indicating that the random permutation of the gene expression matrix and the random edge dropout module contribute effectively to the model’s performance.

In-depth analysis of the model’s spatial representation capabilities reveals that the graph embedding space of ST-GCP can effectively maintain the spatial continuity of tissue structures. As shown in Fig. 2E, UMAP [33] visualization based on Euclidean distance demonstrates that the low-dimensional embeddings generated by ST-GCP clearly distinguish six cortical layers from the WM region. Moreover, the PAGA [42] graphs reveals a linear developmental path from layer 1 to layer 6, and finally to the WM, with gradually changing similarities observed between adjacent layers. Although SEDR, DeepST, and stAA can partially distinguish cortical layers, their trajectories exhibit nonlinear topological features; SpaGCN, STAGATE, and STMSGAL exhibit substantial overlap between different layers (Supplementary Figure S2).

To further demonstrate the importance of random permutation of the gene expression matrix and random edge cropping, we conducted ablation experiments on this dataset. The detailed ARI scores are provided in Table 2. These results demonstrate that both the random permutation of the gene expression matrix and the random edge cropping module are beneficial for the generation of latent embeddings. When these two modules are used in combination, ST-GCP achieves the best performance, as shown in Table 2. The results of these ablation experiments validate the necessity and effectiveness of the ST-GCP framework.

**Table 2:** The ablation results of ST-GCP on the DLPFC dataset.

**Samples methods**	**Slice 151 674**	**Mean**	**Median**
Randomly permutation the gene expression matrix	0.6781	0.5908	0.5739
Random edge dropout	0.6268	0.6075	0.6112
Null	0.5519	0.5217	0.4999
All	0.6819	0.6751	0.6781

### ST-GCP can depict the spatial heterogeneity of human breast cancer tissues

**Figure 3:**
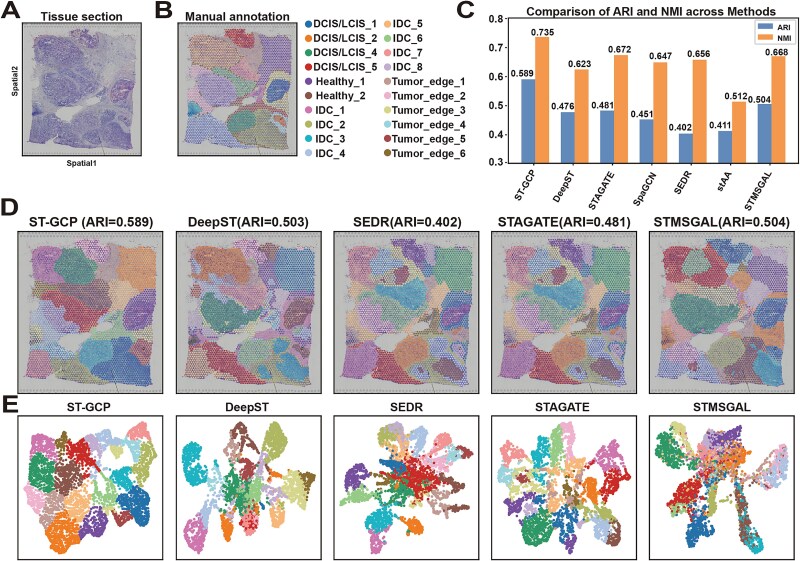
Experimental results of ST-GCP on human breast cancer dataset. (A) Tissue section of this human breast cancer. (B) Manual annotations of this 10x Visium dataset from the SEDR package are divided into 20 regions and classified into four types. (C) Comparison of ARI and NMI metrics for five methods (ST-GCP, DeepST, STAGATE, SEDR, and STMSGAL). (D) Clustering results of six methods on this section are shown. (E) UMAP plots of five methods are displayed.

To demonstrate the generalizability of ST-GCP in cancer tissues, we applied it to a human breast cancer dataset generated using the 10x Visium platform (Fig. 3A). The dataset was manually annotated into 20 regions using the SEDR [22] software package. For ease of analysis, these regions are primarily classified into four morphological types: Healthy, Tumor Edge, Ductal Carcinoma *In Situ*/Lobular Carcinoma *In Situ* (DCIS/LCIS), and Invasive Ductal Carcinoma (IDC), as shown in Fig. 3A. The annotations include two Healthy, five DCIS/LCIS, seven IDC, and six Tumor Edge regions. Notably, DCIS/LCIS regions are typically surrounded by IDC or Tumor Edge, which is clearly shown in the manually annotated ground-truth label map (Fig. 3B). It is worth noting that ST-GCP successfully partitioned 20 spatial regions in this dataset (Fig. 3D), with several clustering results showing high concordance with the manual annotations, including Healthy_1, IDC_2, IDC_3, DCIS/LCIS_1, and Tumor_edge_2.

In the comparative analysis, we evaluated SEDR, SpaGCN, DeepST, STAGATE, stAA, and STMSGAL on the human breast cancer dataset, with their ARI and NMI scores presented in Fig. 3B. ST-GCP demonstrated superior performance compared with all other evaluated methods. Additionally, we compared ST-GCP with three representative methods (DeepST, SEDR, and STMSGAL) that utilized Louvain or Leiden algorithms for downstream clustering. The disorganized tissue partitioning observed with these methods further highlights the advantage of the structure-feature perturbation strategy incorporated in ST-GCP. Among the other three comparative methods (SpaGCN, STAGATE, and stAA), the ARI values were all below 0.5, and the NMI values were below 0.67 (Fig. 3C).

The spatial domain delineations produced by ST-GCP, SEDR, DeepST, STAGATE, and STMSGAL on the human breast cancer dataset (Supplementary Fig. S3) are presented in Fig. 3D. ST-GCP’s clustering output shows strong alignment with the ground-truth annotations. For instance, in the IDC_2 region (Fig. 3A), ST-GCP’s clustering closely matches the ground-truth annotation, whereas DeepST and SEDR divide it into two distinct regions, and STAGATE and STMSGAL further fragment it into several unrelated domains—likely due to their inadequate spatial modeling. Similarly, for the DCIS/LCIS_5 region, ST-GCP achieves a more accurate delineation compared with the four baseline methods, which exhibit notable discrepancies from the ground-truth. These findings further underscore the robustness and effectiveness of ST-GCP.

We also present the UMAP visualizations of the six methods on the human breast cancer dataset (Fig. 3E). The clustering boundaries generated by ST-GCP are relatively distinct, particularly in the regions corresponding to Healthy_1, IDC_2, IDC_3, DCIS/LCIS_1, and Tumor_edge_2, indicating strong spatial discriminative power. In contrast, the visualizations of DeepST, STAGATE, and STMSGAL appear relatively disorganized, suggesting suboptimal performance in spatial clustering. The SEDR results exhibit local compactness, with clustering regions overly concentrated, which compromises the preservation of biological structures. Overall, ST-GCP demonstrates superior performance in spatial clustering resolution and structural preservation, validating the effectiveness of its design.

### ST-GCP supports the parsing of tissue structures in multispatial resolution slide-seqV2 and stereo-seq data

**Figure 4:**
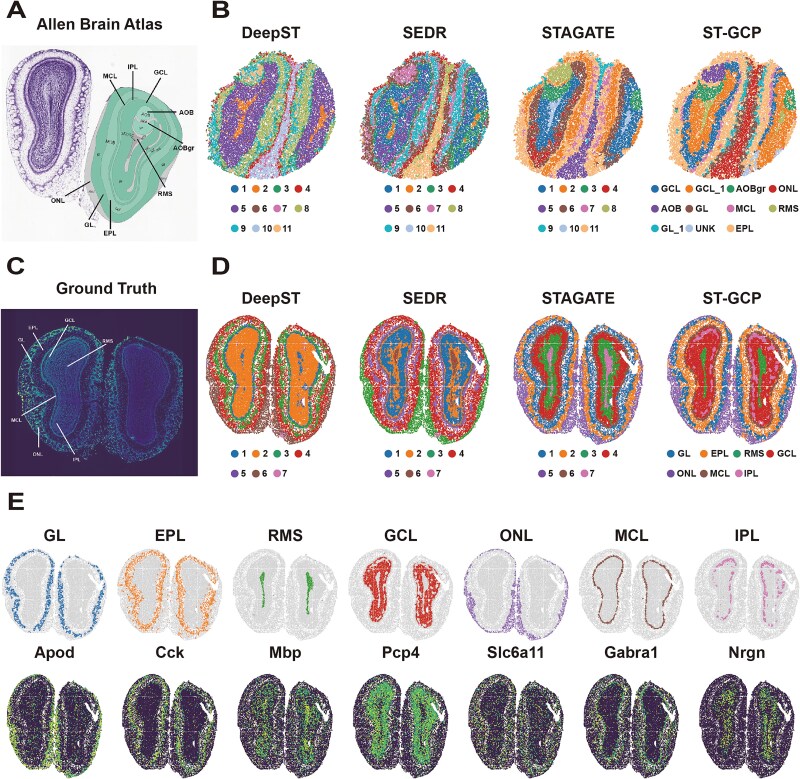
ST-GCP identifies laminar structures in spatial domains of mouse olfactory bulb tissue datasets from slide-seqV2 and stereo-seq platforms. (A) Laminar organization of the mouse olfactory bulb annotated based on the Allen reference atlas. (B) Clustering results of deep, SEDR, STAGATE, and ST-GCP using the mclust method on the slide-seqV2 mouse olfactory bulb tissue section. (C) Laminar organization of the mouse olfactory bulb annotated in the DAPI-stained image generated by stereo-seq. (D) Spatial domains identified by DeepST, SEDR, STAGATE, and ST-GCP using the mclust method in the mouse olfactory bulb stereo-seq data. (E) Visualization of spatial domains identified by ST-GCP and their corresponding marker genes, with the identified domains matching the annotated laminar organization of the mouse olfactory bulb.

We evaluate the performance of ST-GCP in analyzing ST data with different spatial resolutions, particularly its efficacy in identifying tissue laminar structures in mouse olfactory bulb data at varying resolutions. To this end, we apply ST-GCP to mouse olfactory bulb coronal section data analyzed by the Slide-seqV2 platform, which offers a spatial resolution of ~10 μm. Compared with 10x Visium (55 μm), Slide-seqV2 [6] can capture spatial transcriptional information at a near-single-cell scale, characterized by a large number of spots per section (>10 000) but relatively low sequencing depth per spot. The spatial structures identified by ST-GCP in this dataset were highly consistent with the tissue annotations provided by the Allen Reference Atlas (Fig. 4A). With the aid of the mclust algorithm (Fig. 4B), ST-GCP could accurately partition spatial domains such as AOB (the accessory olfactory bulb), AOBgr (the granular layer of the accessory olfactory bulb), GL_1, and UNK. Compared with the laminar structures identified by DeepST, SEDR, and STAGATE, ST-GCP produces clearer spatial domain boundaries. For example, when distinguishing AOBgr from surrounding tissue layers, although DeepST, SEDR, and STAGATE could identify the main laminar structures, there were boundary ambiguities and even overlaps in some regions. This indicates that ST-GCP has more prominent capabilities in perceiving and parsing complex spatial structures under high-resolution scenarios [43].

To further evaluate the capability of ST-GCP in identifying tissue structures, the study selected mouse olfactory bulb tissue with a typical laminar organization as test data, using spatially resolved transcriptomics data based on Stereo-seq technology. This technology achieves subcellular spatial resolution via DNA nanoball patterned array chips, offering a resolution of ~14 μm, enabling the capture of expression features approaching single-cell scale [38]. In the experiment, manual annotations of the multilayered structures in the coronal plane of the mouse olfactory bulb were performed based on DAPI-stained images, including the olfactory nerve layer (ONL), glomerular layer (GL), mitral cell layer (MCL), rostral migratory stream (RMS), granular cell layer (GCL), external plexiform layer (EPL), and internal plexiform layer (IPL) (Fig. 4C). Guided by known anatomical structures, ST-GCP applied the mclust clustering method to partition tissue regions (Fig. 4D). Its clustering results not only effectively divided the tissue regions into seven spatial structural units but also exhibited a high degree of consistency between each cluster and the known tissue layers, fully demonstrating the reliability and accuracy of this method in spatial domain identification. Comparative analysis revealed that the clusters identified by DeepST and SEDR had a lower degree of discrimination for spatial regions, whereas the clusters obtained by STAGATE and ST-GCP through embedding learning better reflected the characteristics of laminar tissues and showed good correspondence with the annotated hierarchical structures. Notably, ST-GCP could clearly identify the continuous RMS tissue structure, and this performance was particularly prominent (Supplementary Fig. S4). To verify the biological rationality of the clustering results, this study introduced the expression patterns of multiple tissue-specific marker genes for validation. The results showed that the tissue structures of GL, RMS, ONL, and MCL in ST-GCP clustering had clear boundaries and were consistent with the expression distributions of mitral cell-related genes (e.g. Apod, Mbp, Slc6a11, and Gabra1). Furthermore, some marker genes (e.g. Cck, Pcp4) exhibited expression overlap between adjacent layers, reflecting the continuity and sharing of cell type distributions across different layers. In summary, ST-GCP not only can effectively identify the tissue partitions of the olfactory bulb but also exhibits spatial resolution capability, providing strong support for structural tissue analysis in high-resolution STs data.

### ST-GCP can parse the similarity between adjacent points in 10x Visium mouse brain data

**Figure 5:**
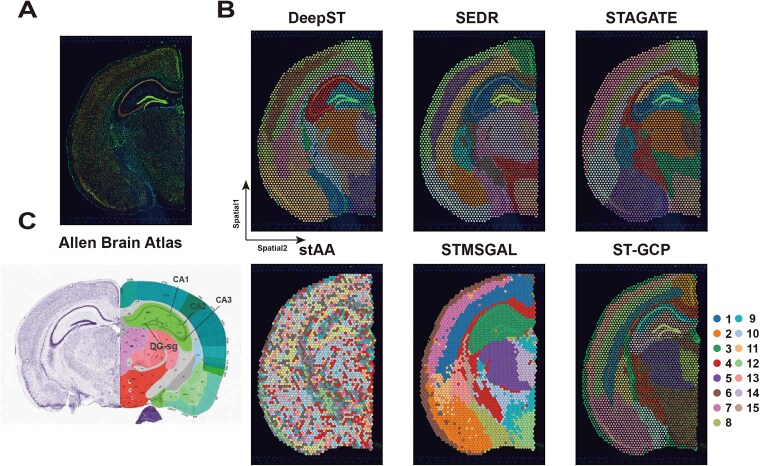
ST-GCP reveals spatial structural domains in the coronal region of the adult mouse brain generated by the 10x Visium platform. (A) Immunofluorescence image of the tissue section after DAPI and anti-NeuN staining. (B) Clustering results of six methods (DeepST, SEDR, STAGATE, stAA, STMSGAL, and ST-GCP) on this dataset, including the results of ST-GCP using mclust and Louvain clustering algorithms. (C) Layered structure of the coronal region of the mouse brain annotated based on the Allen reference atlas.

Next, we evaluated the performance of ST-GCP in complex tissue structures by applying it to STs data of mouse coronal brain sections generated by 10x Visium (Fig. 5A). The manually annotated tissue region labels of this dataset (Fig. 5B) were used to assess the model’s ability to partition spatial structural domains. The spatial domain identification results of DeepST, SEDR, STAGATE, stAA, STMSGAL, and ST-GCP for the tissue section are presented in Fig. 5C**.** Comparative analysis revealed that STMSGAL could preliminarily identify the Cornu Ammonis structure in the hippocampal region but failed to fully recognize finer-grained spatial structures. The spatial clustering results of stAA exhibit ambiguous boundaries between domains and show low consistency with histological annotations. DeepST, SEDR, and STAGATE all effectively detected the typical “cord-like” Cornu Ammonis and “arrow-like” dentate gyrus structures in the hippocampus, yet they showed ambiguity in depicting fine structural domains. In contrast, ST-GCP more clearly distinguished subregional boundaries within the hippocampus and demonstrated higher resolution in the detailed expression of spatial structures (Fig. 5C).

Specifically, based on mclust prior clustering, ST-GCP can successfully separate multiple subregions in the hippocampus, including the CA1, CA2, and CA3 regions of the Ammon’s horn as well as the dentate gyrus region. The boundaries between these regions transition naturally, and the gene expression within each region shows better consistency. Notably, ST-GCP also exhibits excellent discriminatory power in the hierarchical structure of the cerebral cortex, accurately delineating the spatial distribution of multiple cortical layers (e.g. domains 3, 12, and 13). In summary, when faced with ST data characterized by strong cell-type heterogeneity and limited spatial resolution, ST-GCP based on the mclust algorithm enhances the discriminability of spatial domain boundaries in clustering analysis by introducing permutation graph-construction strategies (including gene expression matrix random permutation and random edge dropout), and captures spatial homogeneity within tissues at the microstructural level, providing a novel and refined modeling capability for analysis of complex tissue architectures. Meanwhile, the generation of spatial neighbor networks via Euclidean distance further improves the identification accuracy of structural domains.

We further validated the effectiveness of the random permutation graph-construction strategy in another set of 10x Visium STs data from the mouse hindbrain. The results showed that this module can enhance the ability to recognize fine-grained spatial structural domains. Specifically, ST-GCP successfully revealed the thin layer region of the coronal structure, which was biologically validated by the high expression of Calb1 (structural domain 1) in Supplementary Fig. S5. In contrast, comparative methods such as DeepST, SEDR, STAGATE, stAA and STMSGAL failed to fully recognize this structure, with most methods showing blurred overlaps in the delineation of structural domain boundaries. Additionally, ST-GCP also demonstrated higher resolution in structural domains 1, 6, and 15, which could more closely matched the partitions observed in histological images. In contrast, other methods generally exhibited problems of spatial boundary confusion in structural domain division. For example, the regions identified by STAGATE and SEDR often spanned multiple tissue structures, leading to ambiguous spatial point attribution and difficulty in accurately mapping to histological features. In contrast, the structure-feature perturbation strategy employed by ST-GCP enhanced its sensitivity to local spatial relationships, leading to greater robustness and discriminative power in reconstructing complex brain region structures. The result supports the potential of the permutation graph construction mechanism in capturing microscale tissue heterogeneity.

## Discussion 

Accurate identification of spatial structural domains is essential for understanding tissue architecture and its underlying regulatory mechanisms. In this study, we propose ST-GCP, a graph neural network model that integrates a structure–feature perturbation strategy with gene expression and spatial coordinate information, thereby reducing dependence on labeled STs data and enhancing the utilization of spatial information as well as representation learning effectiveness. Specifically, ST-GCP applies random shuffling of the gene expression matrix in combination with random edge dropout to generate multiview representations, which improves the model’s sensitivity to local spatial structures. Sharing the same adjacency structure across the two views preserves the inherent spatial neighborhood information, ensuring that the underlying spatial relationships between neighboring cells are maintained without requiring additional adjustments to graph connectivity. In addition, maintaining structural consistency helps the model remain robust when handling heterogeneous spatial domains and ensures that spatial neighborhood information is fully utilized in multiview modeling, thereby enhancing the reliability of representation learning and improving the performance of downstream tasks. A spatial neighbor graph is constructed based on Euclidean distance, and a GCN is employed for joint modeling. The model is optimized using reconstruction and contrastive losses, and the learned low-dimensional embeddings can be utilized for downstream analyses such as spatial domain segmentation and trajectory inference.

We conducted a comprehensive evaluation of ST-GCP across multiple ST datasets and benchmarked its performance against seven widely used spatial clustering methods: DeepST, BayesSpace, SEDR, SpaGCN, STAGATE, stAA, and STMSGAL. Overall, ST-GCP demonstrated strong clustering performance across diverse tissue types. It achieved the highest ARI and NMI scores in the majority of slices from the DLPFC dataset, highlighting its robustness and adaptability to complex cortical architectures. Beyond the DLPFC dataset, ST-GCP accurately identified critical spatial domains in mouse brain tissue, olfactory bulbs, and tumor microenvironments, indicating its broad applicability across a range of biological contexts and its resilience to variations in spatial resolution. Moreover, the learned cell embeddings and clustering assignments produced by ST-GCP can be readily applied to downstream analyses such as trajectory inference. To further assess its performance in reconstructing hierarchical tissue structures at single-cell resolution, we applied ST-GCP to an *in situ* hybridization dataset generated using STARmap, which includes expression profiles of 1020 genes across 1207 cells (results detailed in Supplementary Fig. S6). Using expert-curated tissue annotations as the reference standard, ST-GCP achieved the highest clustering accuracy (ARI = 0.563) among six tested methods, with SEDR ranking second (ARI = 0.532). Notably, ST-GCP exhibited a strong ability to capture spatial heterogeneity at the single-cell level, and its clustering outputs showed high concordance with histological annotations. Taken together, these results underscore the versatility and robustness of ST-GCP. We anticipate that its core algorithmic framework can be further adapted to support single-cell analysis tasks in emerging subcellular-resolution technologies, such as osmFISH and PIXEL-seq.

## Conclusion

In summary, ST-GCP is a powerful method based on a structure-feature perturbation mechanism and contrastive consistency, capable of jointly analyzing ST data from different regions. It demonstrates outstanding performance and strong generalizability, efficiently clustering ST data generated from various sequencing platforms, and providing critical support for in-depth ST analyses. Nevertheless, there are still aspects of the model that warrant further optimization. For example, although the current random structure-feature perturbation mechanism helps enhance model robustness, it may unintentionally remove key expression features or disrupt spatial adjacency relationships, potentially affecting clustering accuracy. Additionally, recent methods such as STANDS [44], GAAEST [45], stDCL [46], and MEATRD [47] have shown the potential of leveraging morphological images to capture rich spatial structural information and enhance spatial clustering; however, designing a stable and efficient framework to integrate imaging data with gene expression remains an unresolved challenge. Moreover, ST-GCP currently relies on conventional clustering algorithms for downstream analysis, which may limit its effectiveness when applied to complex tissue architectures or highly heterogeneous samples [48]. Future work could explore incorporating advanced deep clustering methods, graph-based contrastive techniques, or few-shot learning strategies to further improve the model’s accuracy, robustness, and scalability across diverse STs data. Furthermore, incorporating multimodal information and high-performance deep learning models may improve analytical precision, while extending the analysis to 3D perspectives could provide deeper insights into tissue structural organization.

**Key Points**

- We propose feature-level random permutation of the gene expression matrix together with random edge dropout on the spatial neighbor network to create two complementary augmented views of ST data.
- We design a composite loss function that integrates reconstruction and contrastive objectives to jointly optimize structural consistency and latent low-dimensional representations, thereby enhancing the robustness and performance of the ST-GCP framework.
- Extensive experiments on benchmark datasets demonstrate the effectiveness of our approach. Further analysis on unlabeled ST datasets also demonstrates the capability of ST-GCP in identifying spatial domains.

## Supplementary Material

Supplementary_Material_bbaf643

## Data Availability

The data used in this paper are all publicly available. Specifically, the human DLPFC dataset with 12 slices can be obtained from spatialLIBD (http://spatial.libd.org/spatialLIBD/). The 10x Visium human breast cancer dataset can be downloaded from https://support.10xgenomics.com/spatial-gene-expression/datasets/1.0.0/V1_Breast_Cancer_Block_A_Section_1. The annotation file can be found on the SEDR website: https://github.com/JinmiaoChenLab/SEDR_analyses/tree/master/data/BRCA1. The mouse brain coronal dataset of the 10x Visium mouse brain can be obtained from https://www.10xgenomics.com/resources/datasets/adult-mouse-brain-section-1-coronal-stains-dapi-anti-neu-n-1-standard-1-1-0 and the mouse brain posterior dataset of the 10x Visium mouse brain can be obtained from https://www.10xgenomics.com/datasets/mouse-brain-serial-section-1-sagittal-posterior-1-standard-1-1-0. The Slide-seqV2-based mouse olfactory bulb dataset can be obtained from https://portals.broadinstitute.org/single_cell/study/slide-seq-study. The Stereo-seq-based mouse olfactory bulb dataset is accessible on. https://drive.google.com/drive/folders/10lhz5VY7YfvHrtV40MwaqLmWz56U9eBP. The STARmap-based mouse visual cortex dataset is from https://www.dropbox.com/sh/f7ebheru1lbz91s/AADm6D54GSEFXB1feRy6OSASa/visual_1020/20180505_BY3_1kgenes?dl=0&subfolder_nav_tracking=1. Detailed information and publicly accessible source links can be found in the Supplementary Materials.

## Code availability

The code used in this paper is available on GitHub (https://github.com/wyk-wuhan/ST-GCP.git).
