# Supplementary material for: ST-GCP: a graph convolutional network model with contrastive consistency and permutation for spatial transcriptomics
Source: Brief Bioinform. 2025 Dec 5;26(6):bbaf643. doi: 10.1093/bib/bbaf643 (PMC13223589; doi:10.1093/bib/bbaf643)
Supplement: Supplementary_Material_bbaf643 [file supplementary_material_bbaf643.docx]

**Supplementary Figures for**

**ST-GCP: A Graph Convolutional Network Model with Contrastive Consistency and Permutation for Spatial Transcriptomics**


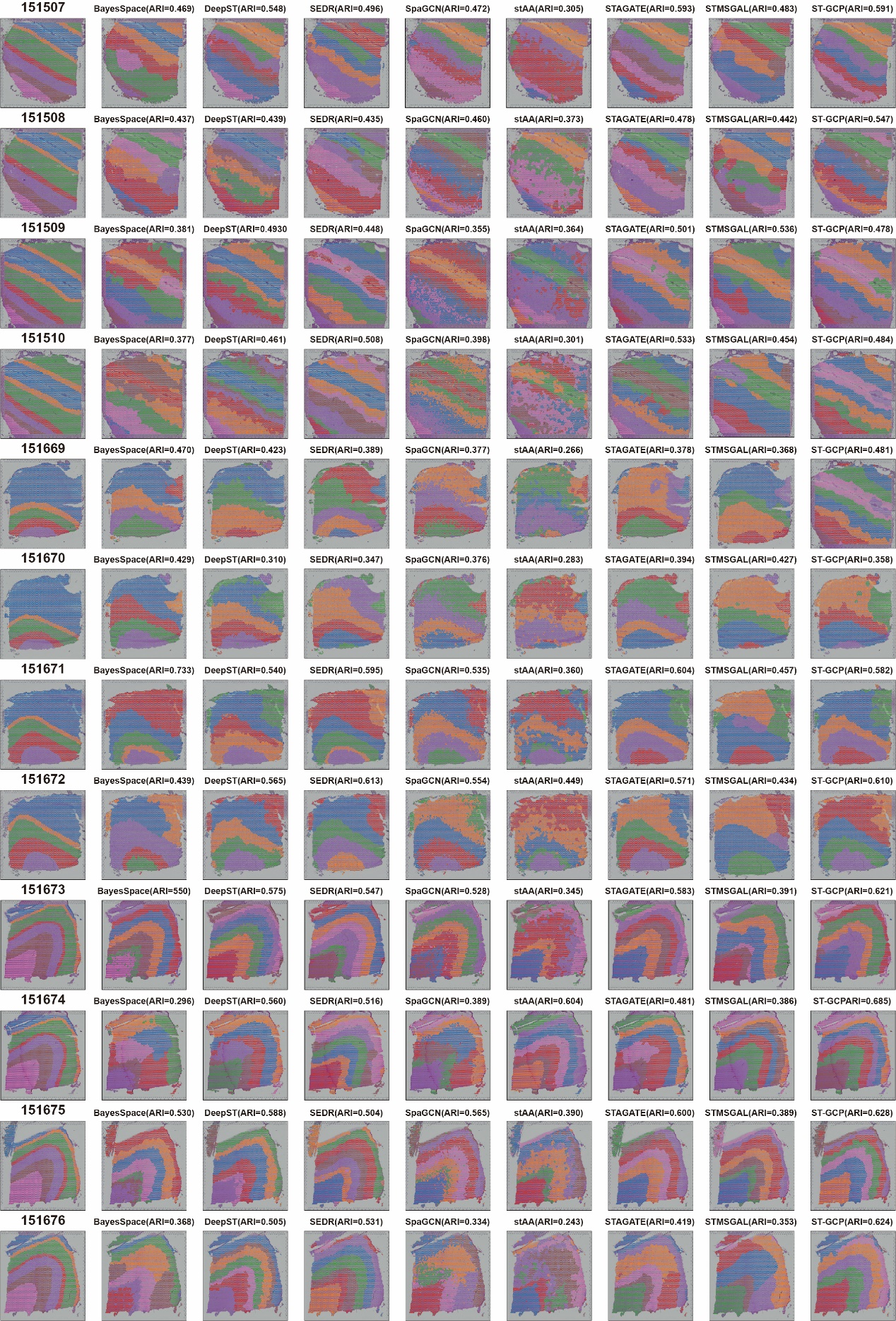


**Supplementary Figure 1.** Clustering performance of eight methods (ST-GCP, SEDR, BayesSpace, SpaGCN, DeepST, STAGATE, stAA, and STMSGAL) on 12 slices of the DLPFC dataset.


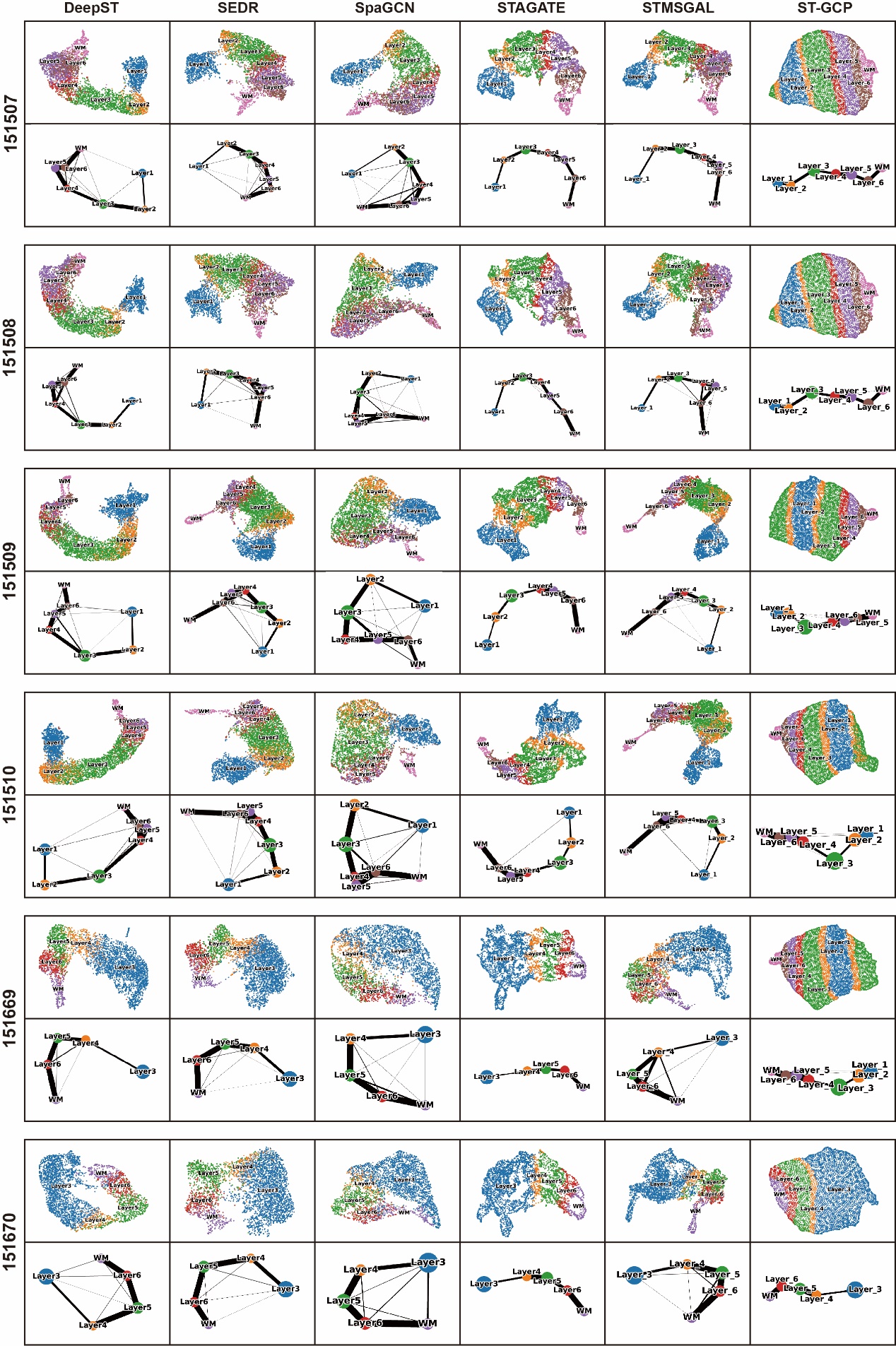


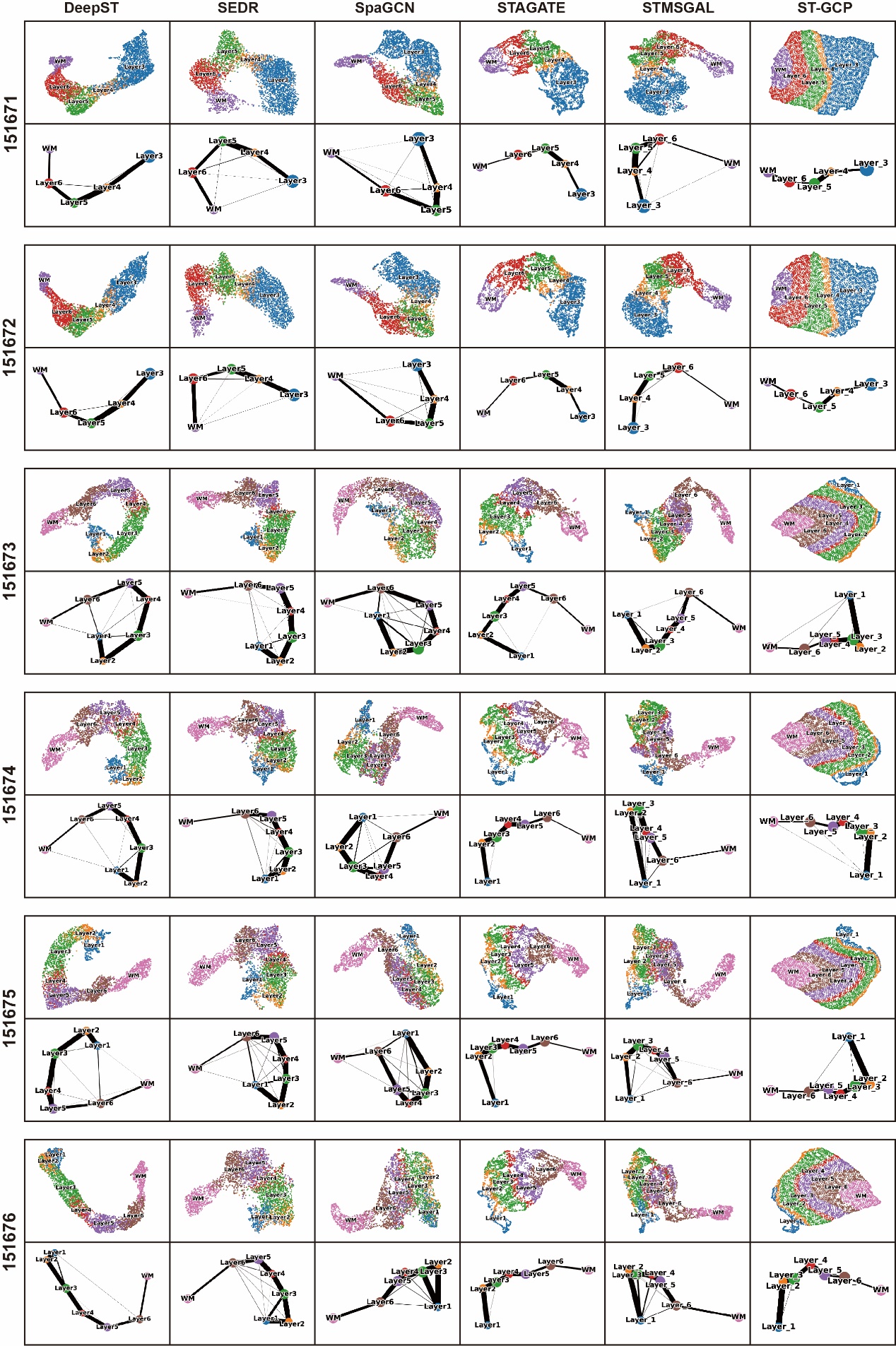


**Supplementary Figure 2**. UMAP visualizations and PAGA graphs generated by of eight methods on 12 slices of the DLPFC dataset.


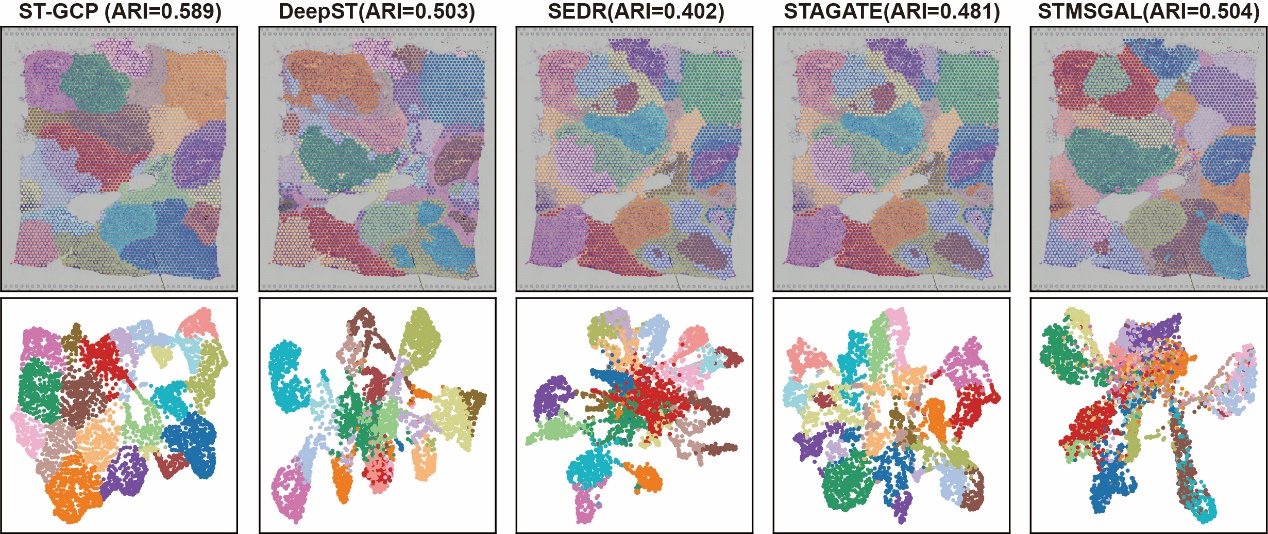


**Supplementary Figure 3.** Clustering results of seven methods (ST-GCP, SEDR, SpaGCN, DeepST, STAGATE, stAA, and STMSGAL) on the human breast cancer dataset.


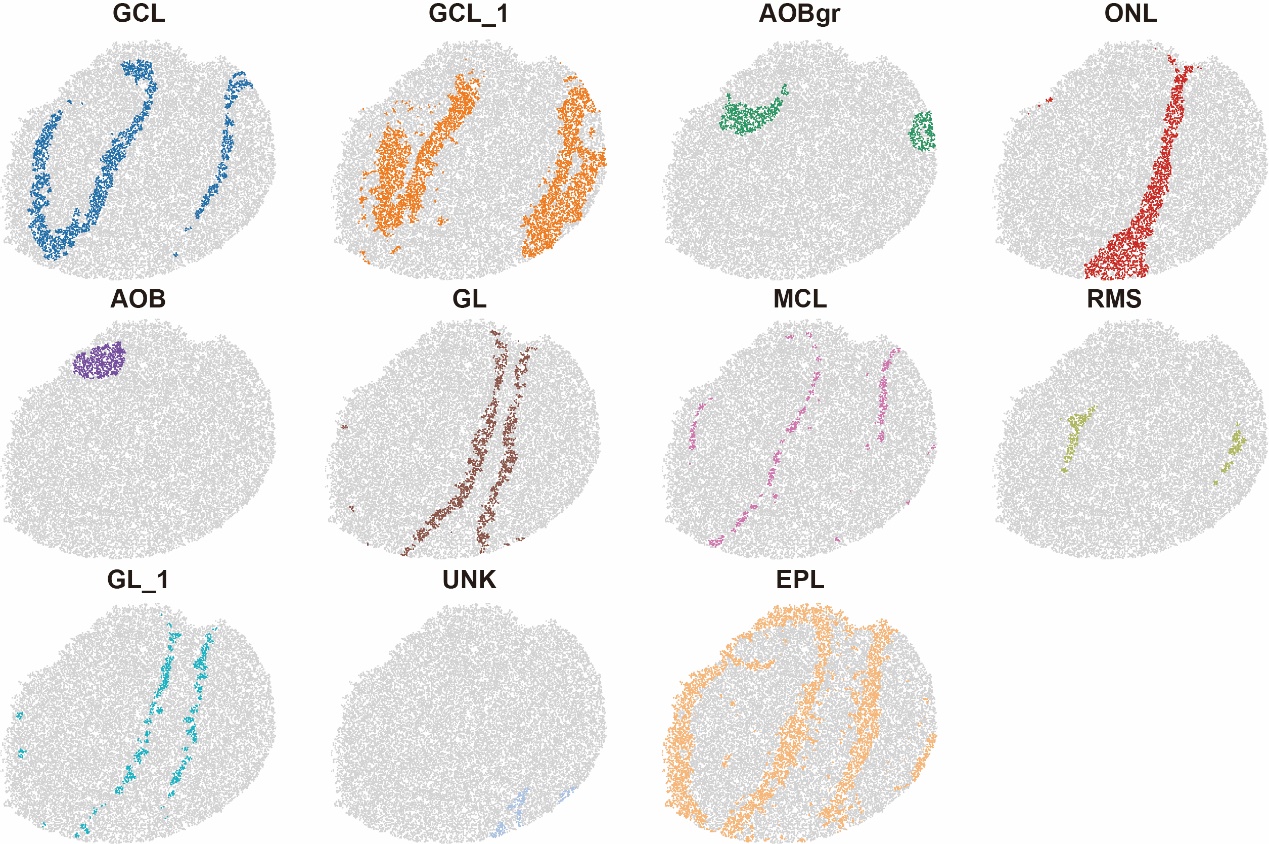


**Supplementary Figure 4.** Spatial domains identified by ST-GCP. The spatial domains were annotated based on the laminar organization presented in the Allen Reference Atlas.


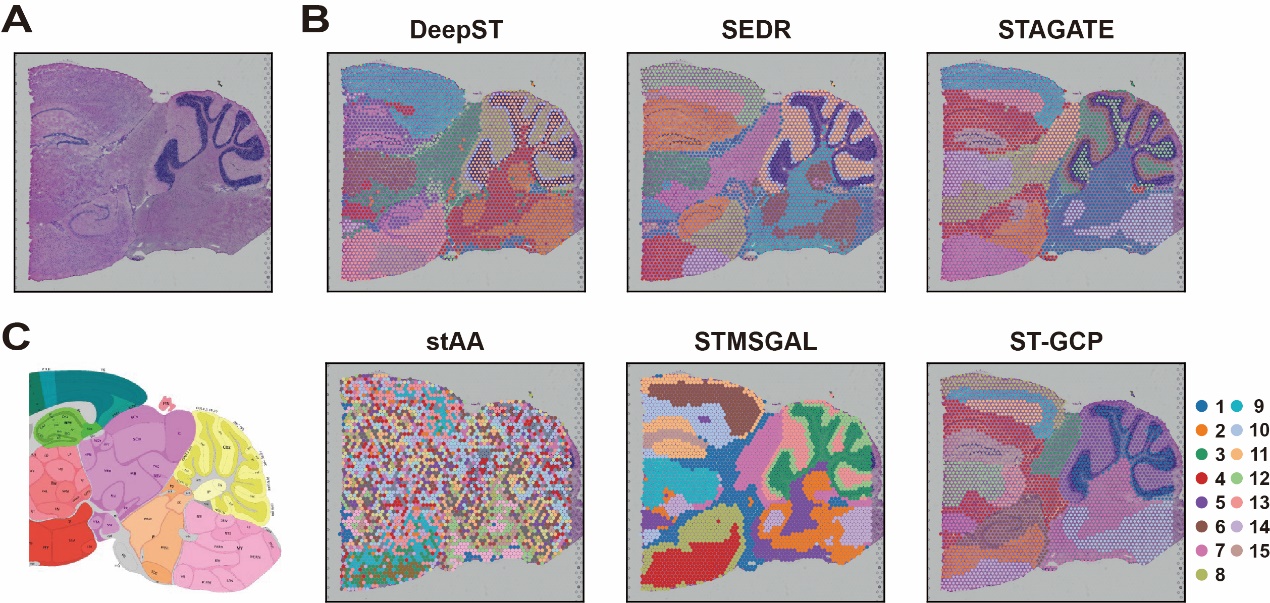


**Supplementary Figure 5.** Clustering results of seven methods (ST-GCP, SEDR, SpaGCN, DeepST, STAGATE, stAA, and STMSGAL) on the 10x Visium mouse posterior brain dataset.


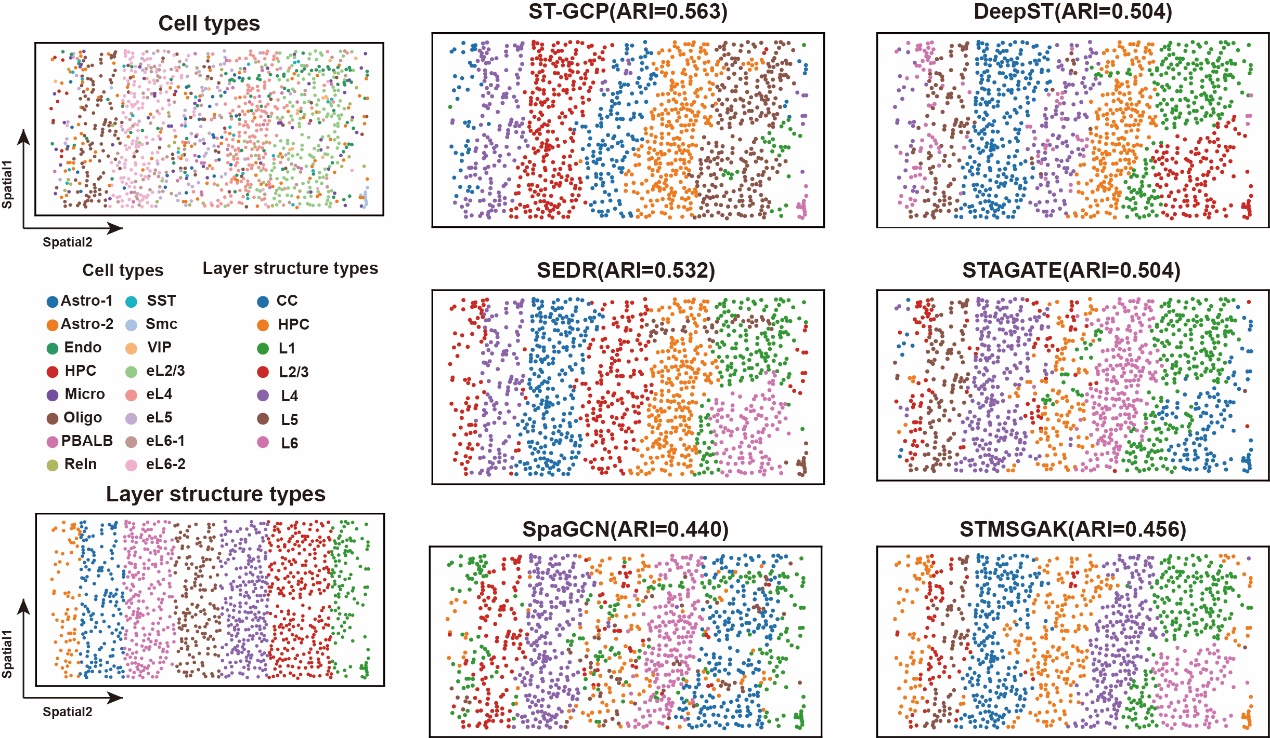


**Supplementary Figure 6.** Clustering results and ground-truth annotations of seven methods (ST-GCP, SEDR, SpaGCN, DeepST, STAGATE, stAA, and STMSGAL) on the STARmap dataset.
